# Characteristics of Esketamine Prescribers Among Medicare Beneficiaries in the United States, 2019-2020

**DOI:** 10.1001/jamanetworkopen.2023.11250

**Published:** 2023-04-27

**Authors:** John L. Havlik, Michael J. Murphy, Nina Gong, Deanna Tang, John H. Krystal

**Affiliations:** 1 Department of Psychiatry, Yale School of Medicine, New Haven, Connecticut; 2 Biological Sciences Division, The University of Chicago, Chicago, Illinois

## Abstract

This cross-sectional study examines the characteristics and geographic distribution of eskatamine prescribers among Medicare beneficiaries in the US from 2019 to 2020.

## Introduction

Esketamine is the first US Food and Drug Administration approved rapid-acting drug for treatment-resistant depression (TRD) and the first novel-mechanism antidepressant in more than 60 years.^1^ Approved on March 5, 2019, esketamine rapidly ameliorates symptoms in the estimated 5% to 6% of individuals with pharmaceutically treated depression who meet criteria for TRD.^2,3^

Geographic and clinician-level patterns of esketamine prescription remain unclear. Ensuring equitable access to new drugs like esketamine is of interest given well-documented rural-urban disparities in access to mental health care and differences in the prevalence of major depressive disorder and other mood disorders between rural and urban populations. Further intersectional disparities may exist, as psychiatric clinicians who only accept self-pay or private insurances may adopt therapies at different rates than those who accept lower-reimbursing public insurances.^4,5^

This cross-sectional study examines early adoption and prescription patterns of esketamine among Medicare prescribers and beneficiaries from 2019 to 2020. The study aims to assess which clinicians have been early adopters of prescribing esketamine to their patients and how these clinicians are geographically distributed.

## Methods

This study followed Strengthening the Reporting of Observational Studies in Epidemiology (STROBE) reporting guideline. This study did not require institutional review board approval as it was based on publicly available information, in accordance with 45 CFR §46. Informed consent was not needed because patient data were deidentified and anonymous.

Information about prescribers, beneficiaries, costs, and counties of esketamine prescriptions were obtained from publicly available Medicare Part D Provider Utilization and Payment Data. The 2013 Rural-Urban Continuum Codes (RUCC) were obtained for counties of prescription from the National Center for Health Statistics. Counties were categorized into metropolitan (RUCC 1-3) or nonmetropolitan (RUCC 4-9). Shapiro-Wilk testing was used to assess normality, with *t* tests used to compare between groups. Linear multivariate regression was used to assess for association of sociodemographic factors with total claims filed. Analyses were 2-tailed with significance set at *P* < .05; analysis was performed using Stata version 17.0 (StataCorp).

## Results

Aggregate cost and claims information from all prescribers submitting claims to Medicare for esketamine are presented in Table 1. Our study subpopulation comprised all 414 unique prescribers submitting more than 10 claims for esketamine to Medicare in 2019 or 2020; of clinicians with available gender data, 225 (35.7%) were female. Of clinicians with available practice location data, 37 (10.6%) practiced in nonmetropolitan areas. Psychiatrists comprised the highest percentage of prescribers each year in metropolitan regions (99 [70.2%] in 2019 and 173 [64.1%] in 2020), but this percentage and the overall percentage of physician prescribers decreased in 2020 (Table 2). Nonpsychiatrist physicians comprised a small proportion of prescribers regardless of geographic area (2020: 17 [5.6%]). Notably, physician assistants, nurse practitioners, and other advanced practice clinicians (APCs) comprised the highest percentage of prescribers each year in nonmetropolitan regions (9 [45.0%] in 2019 and 16 [47.1%] in 2020).

**Table 1.** Trends in the Total Number of Prescribers, Beneficiaries, Claims, and Total Cost of Esketamine in the Medicare Population From 2019 to 2020

Year	Total prescribers, No.	Total claims, No.	Claims per prescriber, mean	Total beneficiaries, No.	Total drug supply days, No.	Total cost, 2022 US dollars, $	Cost per drug supply day, 2022 US dollars, $
2019^a^	448	6173	13.8	431	185 346	8 831 825.03	47.65
2020	684	17 092	25.0	1273	513 018	25 089 316.00	48.91
AAGR, %	52.7	176.9	81.2	195.4	176.8	184.1	2.6

Abbreviation: AAGR, average annual growth rate.

^a^ Esketamine approved March 5, 2019.

**Table 2.** Trends in the Type, Number, and Density of Prescribers Submitting More Than 10 Claims for Esketamine to Medicare from 2019 to 2020 in Metropolitan and Nonmetropolitan Regions

Prescriber	RUCC 1-3			RUCC 4-9			All RUCC		
	2019 No.	2020 No.	AAGR No. (%)	2019 No.	2020 No.	AAGR No. (%)	2019 No.	2020 No.	AAGR No. (%)
**Psychiatrists**									
Total number of prescribers, No. (%)	99 (70.2)	173 (64.1)	74 (74.7)	8 (40.0)	14 (41.2)	6 (75.0)	107 (66.5)	187 (61.5)	80 (74.8)
Total claims	2954	7476	4522 (153.1)	324	813	489 (150.9)	3278	8289	5011 (152.9)
Claims per prescriber, median (IQR)	19 (14-29)	26 (15-52)	7 (36.8)	30.5 (16-58)	48 (28-65)	17.5 (57.4)	19 (14-32)	27 (15-52)	12.0 (63.2)
Claims per 100 000 beneficiaries	16.7	38.7	22.0 (131.7)	12.9	28.0	15.1 (117.1)	16.2	37.3	21.1 (130.2)
**Nonpsychiatrist physicians** ^a^									
Total number of prescribers, No. (%)	7 (5.0)	13 (4.8)	6 (85.7)	3 (15.0)	4 (11.8)	1 (33.3)	10 (6.2)	17 (5.6)	7 (70.0)
Total claims	138	860	722 (523.2)	90	363	273 (303.3)	228	1223	995 (436.4)
Claims per prescriber, median (IQR)	18 (12-26)	36 (18-48)	18 (100.0)	30 (20-40)	61 (13-169)	31.0 (103.3)	21.5 (15-30)	36 (17-109)	14.5 (67.4)
Claims per 100 000 beneficiaries	0.8	4.5	3.7 (462.5)	3.6	12.4	8.8 (244.4)	1.1	5.5	4.4 (400.0)
**NP/PA/other APC**									
Total number of prescribers, No. (%)	35 (24.8)	84 (31.1)	49 (140.0)	9 (45.0)	16 (47.1)	7 (77.8)	44 (27.3)	100 (32.9)	56 (127.3)
Total claims	820	3406	2,586 (315.4)	229	889	660 (288.2)	1049	4295	3246 (309.4)
Claims per prescriber, median (IQR)	20 (13-31)	34.5 (18-48)	14.5 (72.5)	25 (18-34)	34 (23-77)	9.0 (36.0)	20 (14-13.5)	34 (19-50)	14.0 (70.0)
Claims per 100 000 beneficiaries	4.6	17.6	13.0 (282.6)	9.1	30.4	21.3 (234.1)	5.2	19.3	14.1 (271.2)
**All prescribers**									
Total number of prescribers, No. (%)	141 (87.6)	270 (88.8)	129 (91.5)	20 (12.4)	34 (11.2)	14 (70.0)	161 (100.0)	304 (100.0)	143 (88.8)
Prescribers per 100 000 beneficiaries	0.8	1.4	0.6 (75.0)	0.8	1.2	0.4 (51.9)	0.8	1.4	0.6 (75.0)
Total claims	3912	11742	7830 (200.2)	643	2065	1422 (221.2)	4555	13087	8532 (187.3)
Claims per prescriber, median (IQR)	19 (14-28)	29 (16-52)	10 (52.6)	25.5 (18-40)	42.5 (22-80)	17.0 (66.7)	20 (14-31)	30 (17-52)	10.0 (50.0)
Claims per 100 000 beneficiaries	22.1	60.8	38.7 (175.1)	25.5	70.6	45.1 (176.9)	22.5	62.1	39.6 (176.0)

Abbreviations: AAGR, average annual growth rate; APC, advanced practice clinician; NP, nurse practitioner; PA, physician assistant; RUCC, rural-urban continuum code.

^a^ Nonpsychiatrist physicians included those specializing in family practice, anesthesiology, neurology, pediatrics, emergency medicine, internal medicine, pain medicine, hospice/palliative care, and general practice.

Prescribers in metropolitan areas submitted a similar quantity of claims to those in nonmetropolitan areas in 2020 (median [IQR]: metropolitan, 29.0 [16.0-52.0]; nonmetropolitan, 29.0 [16.0-57.0]), and total claims per clinician increased in 2020 (44.1 vs 27.2 in 2019, *P* < .001). Prescriber type, gender, and practice location (metropolitan vs nonmetropolitan) were not significantly associated with total claims.

## Discussion

This cross-sectional study suggests that both the number of prescribers and the frequency of prescriptions for esketamine have increased rapidly, while costs per drug supply day have increased negligibly. This analysis demonstrates similar prescribing behavior regardless of clinician type and practice setting.

While psychiatrists are the primary prescribers of esketamine across metropolitan areas, APCs represent the highest percentage of prescribers in nonmetropolitan areas and are growing as a percentage of prescribers in both metropolitan and nonmetropolitan areas. Few if any comparative analyses exist of new therapy adoption in physicians vs nonphysician prescribers. This study suggests independent APCs are critical early adopters of novel therapies, particularly in rural areas.

This study has limitations. This study only includes prescribing patterns for Medicare-eligible recipients, which excludes clinics that exclusively accept self-pay or private insurance and are administering R,S-ketamine and esketamine for depression management. Furthermore, geographic data are only available for prescribers with more than 10 esketamine claims submitted to Medicare. Supply chain disruptions could have also played a role in prescribing patterns. Nonetheless, this analysis is the first to report adoption patterns of a novel-mechanism antidepressant in this country. These findings may provide a useful benchmark for evaluating the adoption and impact of future therapies for psychiatric conditions.

## Notes

Supplement.
Data Sharing Statement
