# Supplementary material for: Characteristics of Esketamine Prescribers Among Medicare Beneficiaries in the United States, 2019-2020
Source: JAMA Netw Open. 2023 Apr 27;6(4):e2311250. doi: 10.1001/jamanetworkopen.2023.11250 (PMC10140803; doi:10.1001/jamanetworkopen.2023.11250)
Supplement: Supplement. — Data Sharing Statement [file jamanetwopen-e2311250-s001.pdf]

Data Sharing Statement

Havlik. Characteristics of Esketamine Prescribers Among Medicare Beneficiaries in the United States, 2019-2020. *JAMA Netw Open*. Published April 27, 2023.
doi:10.1001/jamanetworkopen.2023.11250

Data
Data available: No

Additional Information
Explanation for why data not available: Already publicly available
